# Supplementary material for: Hot spot prediction in protein-protein interactions by an ensemble system
Source: BMC Syst Biol. 2018 Dec 31;12(Suppl 9):132. doi: 10.1186/s12918-018-0665-8 (PMC6311905; doi:10.1186/s12918-018-0665-8)
Supplement: Supplementary file 2 — All relevant MATLAB code. A simple Matlab implement of our predictor. (ZIP 19,742 kb) [file 12918_2018_665_MOESM2_ESM.zip › Additional file 2/Read ASA/ASA website/dbMPIKT/4BPXA.html]

 NetSurfP - Protein Surface Accessibility and Secondary Structure Predictions

  

|  |  |  |
| --- | --- | --- |
|  |  | NetSurfP - Protein Surface Accessibility and Secondary Structure Predictions  Technical University of Denmark |

  


---

```
# For publication of results, please cite:
# A generic method for assignment of reliability scores applied to solvent accessibility predictions.
# Bent Petersen, Thomas Nordahl Petersen, Pernille Andersen, Morten Nielsen and Claus Lundegaard
# BMC Structural Biology 2009, 9:51 doi:10.1186/1472-6807-9-51
#
# Column 1: Class assignment - B for buried or E for Exposed - Threshold: 25% exposure, but not based on RSA
# Column 2: Amino acid
# Column 3: Sequence name
# Column 4: Amino acid number
# Column 5: Relative Surface Accessibility - RSA
# Column 6: Absolute Surface Accessibility
# Column 7: Z-fit score for RSA prediction
# Column 8: Probability for Alpha-Helix
# Column 9: Probability for Beta-strand
# Column 10: Probability for Coil
E G  4BPXA                  1    0.926  72.884  -1.163   0.003   0.003   0.994
E T  4BPXA                  2    0.622  86.244  -1.363   0.018   0.047   0.935
E S  4BPXA                  3    0.639  74.867  -0.300   0.058   0.017   0.925
B M  4BPXA                  4    0.253  50.585  -0.142   0.181   0.016   0.803
E E  4BPXA                  5    0.560  97.815  -0.043   0.115   0.016   0.868
E T  4BPXA                  6    0.478  66.257  -0.295   0.181   0.016   0.803
E F  4BPXA                  7    0.343  68.940  -0.137   0.181   0.016   0.803
E D  4BPXA                  8    0.395  56.977  -0.495   0.115   0.016   0.868
E P  4BPXA                  9    0.472  67.048   0.195   0.622   0.015   0.363
E T  4BPXA                 10    0.477  66.104  -1.241   0.522   0.016   0.462
E E  4BPXA                 11    0.305  53.301   0.619   0.430   0.016   0.555
B L  4BPXA                 12    0.084  15.435   0.913   0.622   0.015   0.363
E P  4BPXA                 13    0.419  59.442   1.054   0.923   0.002   0.076
E E  4BPXA                 14    0.603 105.292   0.127   0.970   0.001   0.030
B L  4BPXA                 15    0.183  33.489   0.661   0.970   0.001   0.030
B L  4BPXA                 16    0.033   6.097   0.683   0.970   0.001   0.030
E K  4BPXA                 17    0.327  67.264   1.655   0.988   0.000   0.012
E L  4BPXA                 18    0.464  84.903   1.409   0.970   0.001   0.030
B Y  4BPXA                 19    0.073  15.643   0.850   0.970   0.001   0.030
B Y  4BPXA                 20    0.059  12.608   0.583   0.923   0.002   0.076
E R  4BPXA                 21    0.512 117.340   1.021   0.858   0.002   0.139
E R  4BPXA                 22    0.554 126.958   0.353   0.694   0.003   0.303
B L  4BPXA                 23    0.240  43.871  -0.312   0.406   0.004   0.590
B F  4BPXA                 24    0.127  25.569   0.325   0.109   0.005   0.886
E P  4BPXA                 25    0.505  71.688  -0.260   0.058   0.017   0.925
B Y  4BPXA                 26    0.239  51.096  -0.446   0.181   0.016   0.803
E S  4BPXA                 27    0.532  62.350   0.316   0.339   0.016   0.645
E Q  4BPXA                 28    0.412  73.583   0.271   0.455   0.046   0.498
B Y  4BPXA                 29    0.129  27.631  -0.302   0.561   0.047   0.393
B Y  4BPXA                 30    0.196  41.885   0.228   0.502   0.102   0.396
E R  4BPXA                 31    0.474 108.638   1.134   0.605   0.105   0.290
B W  4BPXA                 32    0.117  28.114  -0.483   0.660   0.049   0.291
B L  4BPXA                 33    0.165  30.285  -0.898   0.561   0.047   0.393
E N  4BPXA                 34    0.545  79.788  -1.030   0.354   0.048   0.598
B Y  4BPXA                 35    0.286  61.033   0.153   0.115   0.016   0.868
E G  4BPXA                 36    0.606  47.716  -1.719   0.058   0.017   0.925
E G  4BPXA                 37    0.537  42.230  -1.881   0.018   0.019   0.964
E V  4BPXA                 38    0.576  88.531  -1.188   0.018   0.019   0.964
E I  4BPXA                 39    0.360  66.619  -0.170   0.018   0.019   0.964
E K  4BPXA                 40    0.454  93.347  -0.163   0.058   0.017   0.925
E N  4BPXA                 41    0.642  93.974   0.301   0.058   0.017   0.925
E Y  4BPXA                 42    0.306  65.349   0.392   0.053   0.043   0.903
B F  4BPXA                 43    0.052  10.477   0.346   0.052   0.084   0.864
E Q  4BPXA                 44    0.520  92.890   1.317   0.056   0.142   0.802
E H  4BPXA                 45    0.346  62.974   0.835   0.021   0.279   0.699
B R  4BPXA                 46    0.078  17.816   1.044   0.022   0.552   0.426
B E  4BPXA                 47    0.042   7.268  -0.769   0.003   0.718   0.279
B F  4BPXA                 48    0.041   8.189   0.215   0.018   0.846   0.136
B S  4BPXA                 49    0.019   2.203   0.323   0.011   0.918   0.071
B F  4BPXA                 50    0.034   6.744   0.335   0.011   0.918   0.071
B T  4BPXA                 51    0.096  13.371   0.240   0.018   0.846   0.136
B L  4BPXA                 52    0.140  25.707   0.611   0.004   0.616   0.381
E K  4BPXA                 53    0.375  77.137  -0.141   0.004   0.197   0.799
E D  4BPXA                 54    0.663  95.610  -1.490   0.018   0.019   0.964
E D  4BPXA                 55    0.429  61.761  -1.250   0.018   0.047   0.935
B I  4BPXA                 56    0.195  36.075  -0.623   0.021   0.451   0.528
B Y  4BPXA                 57    0.134  28.700   0.017   0.023   0.655   0.322
B I  4BPXA                 58    0.158  29.193   0.954   0.087   0.683   0.230
B R  4BPXA                 59    0.092  21.045  -0.015   0.079   0.592   0.329
B Y  4BPXA                 60    0.168  35.795  -0.127   0.074   0.484   0.442
B Q  4BPXA                 61    0.195  34.773   0.869   0.069   0.386   0.545
B S  4BPXA                 62    0.166  19.408  -0.616   0.022   0.359   0.619
B F  4BPXA                 63    0.078  15.554   0.112   0.020   0.205   0.775
E N  4BPXA                 64    0.533  78.060   0.404   0.005   0.045   0.951
E N  4BPXA                 65    0.365  53.495   0.377   0.018   0.019   0.964
E Q  4BPXA                 66    0.298  53.187  -0.007   0.923   0.002   0.076
E S  4BPXA                 67    0.618  72.371   0.336   0.923   0.002   0.076
E D  4BPXA                 68    0.309  44.527   1.764   0.923   0.002   0.076
B L  4BPXA                 69    0.024   4.413   1.100   0.970   0.001   0.030
E E  4BPXA                 70    0.257  44.985   1.710   0.970   0.001   0.030
E K  4BPXA                 71    0.524 107.746   1.745   0.970   0.001   0.030
B E  4BPXA                 72    0.222  38.696   1.594   0.970   0.001   0.030
B M  4BPXA                 73    0.020   4.022   0.821   0.970   0.001   0.030
E Q  4BPXA                 74    0.295  52.758   1.368   0.970   0.001   0.030
E A  4BPXA                 75    0.711  78.319   0.845   0.923   0.002   0.076
E A  4BPXA                 76    0.331  36.432   1.513   0.694   0.003   0.303
B N  4BPXA                 77    0.127  18.578   1.054   0.109   0.005   0.886
B P  4BPXA                 78    0.156  22.151  -0.892   0.018   0.019   0.964
E Y  4BPXA                 79    0.351  74.902  -0.065   0.018   0.047   0.935
E K  4BPXA                 80    0.269  55.354   0.758   0.021   0.451   0.528
B I  4BPXA                 81    0.035   6.531   0.182   0.002   0.816   0.182
B D  4BPXA                 82    0.071  10.246  -0.965   0.018   0.846   0.136
B I  4BPXA                 83    0.090  16.632  -0.458   0.021   0.756   0.223
B G  4BPXA                 84    0.060   4.722  -0.574   0.022   0.552   0.426
B A  4BPXA                 85    0.090   9.918  -0.265   0.021   0.279   0.699
B V  4BPXA                 86    0.062   9.529  -0.834   0.066   0.296   0.638
B Y  4BPXA                 87    0.211  45.069   0.186   0.066   0.296   0.638
B S  4BPXA                 88    0.270  31.632  -0.951   0.064   0.216   0.721
E H  4BPXA                 89    0.445  80.945   0.281   0.056   0.142   0.802
E R  4BPXA                 90    0.461 105.569   1.119   0.052   0.084   0.864
B P  4BPXA                 91    0.111  15.751  -1.773   0.115   0.016   0.868
E N  4BPXA                 92    0.653  95.658   0.026   0.181   0.016   0.803
E Q  4BPXA                 93    0.489  87.264   0.449   0.257   0.016   0.727
E H  4BPXA                 94    0.501  91.223   0.651   0.257   0.016   0.727
E N  4BPXA                 95    0.677  99.084   0.140   0.184   0.043   0.773
B T  4BPXA                 96    0.275  38.087  -0.452   0.268   0.043   0.689
E V  4BPXA                 97    0.479  73.592  -0.688   0.268   0.043   0.689
E K  4BPXA                 98    0.611 125.765  -0.204   0.354   0.048   0.598
E L  4BPXA                 99    0.456  83.549  -0.763   0.268   0.043   0.689
E G  4BPXA                100    0.504  39.696  -1.630   0.268   0.043   0.689
E A  4BPXA                101    0.353  38.857  -1.256   0.191   0.086   0.723
B F  4BPXA                102    0.257  51.560  -0.219   0.113   0.087   0.800
E Q  4BPXA                103    0.440  78.513   0.227   0.113   0.087   0.800
B A  4BPXA                104    0.185  20.420  -0.114   0.118   0.150   0.732
E Q  4BPXA                105    0.262  46.811   0.593   0.125   0.227   0.648
E E  4BPXA                106    0.410  71.610  -0.054   0.118   0.150   0.732
B K  4BPXA                107    0.205  42.251   0.650   0.056   0.142   0.802
B E  4BPXA                108    0.148  25.838   0.267   0.066   0.296   0.638
B L  4BPXA                109    0.031   5.749   0.834   0.021   0.756   0.223
B V  4BPXA                110    0.042   6.440   0.341   0.018   0.846   0.136
B F  4BPXA                111    0.031   6.222   0.732   0.018   0.846   0.136
B D  4BPXA                112    0.077  11.110  -0.236   0.023   0.655   0.322
B I  4BPXA                113    0.045   8.417   1.235   0.005   0.336   0.660
B D  4BPXA                114    0.197  28.445   0.232   0.004   0.197   0.799
B M  4BPXA                115    0.085  16.948   0.454   0.018   0.047   0.935
E T  4BPXA                116    0.481  66.742   0.300   0.018   0.047   0.935
E D  4BPXA                117    0.284  40.939   0.115   0.053   0.043   0.903
B Y  4BPXA                118    0.138  29.576   0.405   0.053   0.043   0.903
E D  4BPXA                119    0.556  80.048  -0.811   0.058   0.017   0.925
E D  4BPXA                120    0.721 103.939  -0.428   0.018   0.019   0.964
B V  4BPXA                121    0.155  23.900  -0.479   0.058   0.017   0.925
E R  4BPXA                122    0.399  91.371   0.265   0.053   0.043   0.903
E R  4BPXA                123    0.354  81.089   0.480   0.053   0.043   0.903
B C  4BPXA                124    0.050   7.020  -1.198   0.053   0.043   0.903
B C  4BPXA                125    0.186  26.157  -0.913   0.058   0.017   0.925
E S  4BPXA                126    0.540  63.229  -1.220   0.058   0.017   0.925
E S  4BPXA                127    0.478  55.986  -1.229   0.246   0.004   0.750
E A  4BPXA                128    0.464  51.188  -0.606   0.502   0.002   0.495
E D  4BPXA                129    0.605  87.224   1.072   0.694   0.003   0.303
B I  4BPXA                130    0.046   8.473   0.057   0.858   0.002   0.139
B C  4BPXA                131    0.031   4.296   0.126   0.858   0.002   0.139
E P  4BPXA                132    0.587  83.253   1.257   0.970   0.001   0.030
E K  4BPXA                133    0.431  88.677   2.279   0.970   0.001   0.030
B C  4BPXA                134    0.015   2.106   1.448   0.970   0.001   0.030
B W  4BPXA                135    0.092  22.198   1.320   0.970   0.001   0.030
E T  4BPXA                136    0.510  70.709   1.324   0.970   0.001   0.030
B L  4BPXA                137    0.199  36.364   1.149   0.988   0.000   0.012
B M  4BPXA                138    0.016   3.142   1.135   0.988   0.000   0.012
E T  4BPXA                139    0.239  33.122   1.320   0.988   0.000   0.012
E M  4BPXA                140    0.280  55.968   1.309   0.988   0.000   0.012
B A  4BPXA                141    0.049   5.389   0.464   0.970   0.001   0.030
B I  4BPXA                142    0.015   2.738   1.404   0.970   0.001   0.030
E R  4BPXA                143    0.337  77.196   2.017   0.970   0.001   0.030
B I  4BPXA                144    0.167  30.895   1.139   0.970   0.001   0.030
B I  4BPXA                145    0.014   2.590   1.703   0.970   0.001   0.030
B D  4BPXA                146    0.123  17.753   0.209   0.970   0.001   0.030
E R  4BPXA                147    0.406  92.860   1.424   0.970   0.001   0.030
B A  4BPXA                148    0.048   5.290   0.628   0.923   0.002   0.076
B L  4BPXA                149    0.020   3.717   1.366   0.858   0.002   0.139
E K  4BPXA                150    0.323  66.359   1.261   0.782   0.003   0.216
E E  4BPXA                151    0.638 111.528   0.167   0.600   0.003   0.397
B D  4BPXA                152    0.259  37.279  -0.455   0.406   0.004   0.590
B F  4BPXA                153    0.087  17.521  -0.072   0.109   0.005   0.886
E G  4BPXA                154    0.406  31.944  -0.099   0.018   0.019   0.964
B F  4BPXA                155    0.093  18.645   0.146   0.018   0.019   0.964
E K  4BPXA                156    0.599 123.194   1.052   0.018   0.047   0.935
E H  4BPXA                157    0.384  69.886   0.503   0.004   0.197   0.799
B R  4BPXA                158    0.059  13.580   0.771   0.003   0.718   0.279
B L  4BPXA                159    0.140  25.561   0.893   0.001   0.900   0.099
B W  4BPXA                160    0.051  12.265   0.552   0.001   0.959   0.040
B V  4BPXA                161    0.047   7.162   0.845   0.001   0.959   0.040
B Y  4BPXA                162    0.041   8.847   0.361   0.001   0.900   0.099
B S  4BPXA                163    0.103  12.025  -0.377   0.004   0.616   0.381
B G  4BPXA                164    0.118   9.255  -1.814   0.004   0.085   0.910
E R  4BPXA                165    0.308  70.646  -0.558   0.005   0.045   0.951
B R  4BPXA                166    0.192  43.968  -0.697   0.004   0.197   0.799
B G  4BPXA                167    0.046   3.644  -2.560   0.003   0.718   0.279
B V  4BPXA                168    0.048   7.408   0.278   0.002   0.816   0.182
B H  4BPXA                169    0.044   8.076   0.455   0.001   0.900   0.099
B C  4BPXA                170    0.016   2.289   1.446   0.001   0.959   0.040
B W  4BPXA                171    0.104  24.964   1.086   0.001   0.900   0.099
B V  4BPXA                172    0.024   3.720   0.665   0.002   0.816   0.182
B C  4BPXA                173    0.129  18.168   1.368   0.005   0.336   0.660
B D  4BPXA                174    0.207  29.858   0.213   0.184   0.043   0.773
E E  4BPXA                175    0.698 121.871   0.598   0.600   0.003   0.397
E S  4BPXA                176    0.485  56.854   1.042   0.600   0.003   0.397
B V  4BPXA                177    0.047   7.178  -0.411   0.622   0.015   0.363
E R  4BPXA                178    0.374  85.738   0.790   0.430   0.016   0.555
E K  4BPXA                179    0.636 130.763   1.391   0.181   0.016   0.803
B L  4BPXA                180    0.094  17.138   0.190   0.053   0.005   0.942
E S  4BPXA                181    0.557  65.280   0.801   0.016   0.005   0.979
E S  4BPXA                182    0.591  69.218   1.114   0.858   0.002   0.139
E A  4BPXA                183    0.728  80.182   0.708   0.923   0.002   0.076
E V  4BPXA                184    0.265  40.807   0.558   0.970   0.001   0.030
B R  4BPXA                185    0.091  20.747   0.313   0.970   0.001   0.030
E S  4BPXA                186    0.390  45.743   1.162   0.970   0.001   0.030
E G  4BPXA                187    0.292  22.988   1.197   0.970   0.001   0.030
B I  4BPXA                188    0.027   5.088   0.651   0.975   0.003   0.022
B V  4BPXA                189    0.062   9.606   0.477   0.938   0.007   0.055
E E  4BPXA                190    0.436  76.204   0.922   0.879   0.010   0.111
B Y  4BPXA                191    0.100  21.306   0.918   0.879   0.010   0.111
B L  4BPXA                192    0.050   9.063   0.476   0.831   0.044   0.125
E S  4BPXA                193    0.388  45.438   0.723   0.701   0.107   0.192
B L  4BPXA                194    0.201  36.785  -0.966   0.453   0.248   0.299
B V  4BPXA                195    0.193  29.618  -0.067   0.216   0.235   0.548
E K  4BPXA                196    0.425  87.443  -0.679   0.118   0.150   0.732
B G  4BPXA                197    0.310  24.421  -1.555   0.052   0.084   0.864
E G  4BPXA                198    0.470  36.997  -1.057   0.113   0.043   0.844
E Q  4BPXA                199    0.573 102.391  -1.039   0.184   0.043   0.773
E D  4BPXA                200    0.538  77.555  -1.278   0.191   0.086   0.723
E V  4BPXA                201    0.325  49.999  -0.628   0.199   0.152   0.649
E K  4BPXA                202    0.467  96.041  -0.418   0.216   0.235   0.548
E K  4BPXA                203    0.524 107.725  -0.273   0.135   0.317   0.548
E K  4BPXA                204    0.435  89.541  -0.410   0.069   0.386   0.545
B V  4BPXA                205    0.187  28.665  -0.971   0.074   0.484   0.442
E H  4BPXA                206    0.482  87.603  -1.001   0.069   0.386   0.545
B L  4BPXA                207    0.253  46.251  -1.069   0.056   0.142   0.802
E S  4BPXA                208    0.533  62.526  -1.343   0.053   0.043   0.903
E E  4BPXA                209    0.566  98.793  -1.288   0.053   0.043   0.903
E K  4BPXA                210    0.468  96.247  -1.680   0.058   0.017   0.925
B I  4BPXA                211    0.219  40.533  -1.364   0.058   0.017   0.925
B H  4BPXA                212    0.192  34.852  -0.578   0.053   0.005   0.942
E P  4BPXA                213    0.365  51.794  -0.579   0.600   0.003   0.397
B F  4BPXA                214    0.177  35.484  -0.455   0.802   0.014   0.185
B I  4BPXA                215    0.056  10.379  -0.086   0.923   0.002   0.076
E R  4BPXA                216    0.378  86.562   0.689   0.923   0.002   0.076
E K  4BPXA                217    0.354  72.900   0.379   0.923   0.002   0.076
B S  4BPXA                218    0.038   4.418  -1.181   0.923   0.002   0.076
B I  4BPXA                219    0.088  16.243   0.626   0.923   0.002   0.076
E N  4BPXA                220    0.532  77.943   1.236   0.923   0.002   0.076
B I  4BPXA                221    0.179  33.022   1.043   0.970   0.001   0.030
B I  4BPXA                222    0.025   4.625   0.612   0.923   0.002   0.076
B K  4BPXA                223    0.235  48.257   0.990   0.923   0.002   0.076
E K  4BPXA                224    0.557 114.616   0.729   0.858   0.002   0.139
B Y  4BPXA                225    0.171  36.564   0.682   0.782   0.003   0.216
B F  4BPXA                226    0.041   8.229  -0.456   0.694   0.003   0.303
E E  4BPXA                227    0.400  69.863   0.299   0.802   0.014   0.185
E E  4BPXA                228    0.602 105.134   0.066   0.802   0.014   0.185
B Y  4BPXA                229    0.228  48.617   0.736   0.802   0.014   0.185
B A  4BPXA                230    0.071   7.846   0.155   0.717   0.014   0.269
B L  4BPXA                231    0.216  39.513   0.282   0.717   0.014   0.269
E V  4BPXA                232    0.539  82.798   0.311   0.717   0.014   0.269
E N  4BPXA                233    0.428  62.615  -0.730   0.522   0.016   0.462
E Q  4BPXA                234    0.351  62.760  -0.036   0.430   0.016   0.555
E D  4BPXA                235    0.579  83.463  -0.681   0.430   0.016   0.555
B I  4BPXA                236    0.235  43.401  -0.312   0.339   0.016   0.645
B L  4BPXA                237    0.118  21.551  -1.694   0.181   0.016   0.803
E E  4BPXA                238    0.583 101.815  -0.516   0.109   0.005   0.886
E N  4BPXA                239    0.473  69.233  -0.181   0.109   0.005   0.886
E K  4BPXA                240    0.459  94.313  -0.230   0.694   0.003   0.303
E E  4BPXA                241    0.700 122.307   0.704   0.782   0.003   0.216
E S  4BPXA                242    0.358  41.922   1.175   0.782   0.003   0.216
B W  4BPXA                243    0.047  11.303   0.636   0.970   0.001   0.030
E D  4BPXA                244    0.510  73.534   1.132   0.970   0.001   0.030
E K  4BPXA                245    0.451  92.874   1.649   0.923   0.002   0.076
B I  4BPXA                246    0.051   9.361  -0.040   0.923   0.002   0.076
B L  4BPXA                247    0.118  21.587  -0.876   0.802   0.014   0.185
E A  4BPXA                248    0.631  69.547   1.140   0.600   0.003   0.397
E L  4BPXA                249    0.307  56.212   0.549   0.406   0.004   0.590
B V  4BPXA                250    0.129  19.797   0.998   0.115   0.016   0.868
E P  4BPXA                251    0.443  62.890   0.073   0.502   0.002   0.495
E E  4BPXA                252    0.700 122.342   0.520   0.923   0.002   0.076
E T  4BPXA                253    0.452  62.651   0.539   0.923   0.002   0.076
B I  4BPXA                254    0.060  11.063   0.138   0.970   0.001   0.030
B H  4BPXA                255    0.192  34.888   1.118   0.970   0.001   0.030
E D  4BPXA                256    0.565  81.431   1.403   0.988   0.000   0.012
E E  4BPXA                257    0.292  50.995   1.640   0.988   0.000   0.012
B L  4BPXA                258    0.036   6.537   0.311   0.970   0.001   0.030
E Q  4BPXA                259    0.339  60.599   1.393   0.970   0.001   0.030
E Q  4BPXA                260    0.608 108.553   0.844   0.923   0.002   0.076
B S  4BPXA                261    0.265  31.058   0.222   0.782   0.003   0.216
B F  4BPXA                262    0.088  17.581  -0.938   0.600   0.003   0.397
E Q  4BPXA                263    0.523  93.390   0.481   0.502   0.002   0.495
E K  4BPXA                264    0.595 122.474  -0.946   0.321   0.003   0.675
E S  4BPXA                265    0.446  52.330  -1.518   0.109   0.005   0.886
E H  4BPXA                266    0.532  96.680  -1.140   0.109   0.005   0.886
E N  4BPXA                267    0.467  68.413  -1.152   0.109   0.005   0.886
B S  4BPXA                268    0.191  22.409  -1.867   0.321   0.003   0.675
E L  4BPXA                269    0.511  93.582  -0.575   0.694   0.003   0.303
E Q  4BPXA                270    0.509  90.854   0.160   0.622   0.015   0.363
B R  4BPXA                271    0.175  39.983   0.371   0.802   0.014   0.185
B W  4BPXA                272    0.084  20.178   0.558   0.923   0.002   0.076
E E  4BPXA                273    0.580 101.256   1.013   0.970   0.001   0.030
E H  4BPXA                274    0.242  43.983   1.083   0.970   0.001   0.030
B L  4BPXA                275    0.018   3.369   1.146   0.970   0.001   0.030
E K  4BPXA                276    0.339  69.815   1.478   0.970   0.001   0.030
E K  4BPXA                277    0.574 118.031   1.564   0.970   0.001   0.030
B V  4BPXA                278    0.140  21.472   0.531   0.970   0.001   0.030
B A  4BPXA                279    0.044   4.849  -0.081   0.923   0.002   0.076
E S  4BPXA                280    0.630  73.859   1.075   0.923   0.002   0.076
E R  4BPXA                281    0.561 128.583   0.447   0.858   0.002   0.139
B Y  4BPXA                282    0.141  30.132   0.180   0.782   0.003   0.216
E Q  4BPXA                283    0.412  73.655  -0.289   0.694   0.003   0.303
E N  4BPXA                284    0.627  91.807   0.715   0.717   0.014   0.269
E N  4BPXA                285    0.462  67.578  -0.768   0.622   0.015   0.363
E I  4BPXA                286    0.299  55.296  -0.979   0.430   0.016   0.555
E K  4BPXA                287    0.512 105.318  -0.450   0.522   0.016   0.462
E N  4BPXA                288    0.399  58.355  -0.506   0.717   0.014   0.269
E D  4BPXA                289    0.340  48.980  -1.114   0.802   0.014   0.185
E K  4BPXA                290    0.430  88.451   0.031   0.802   0.014   0.185
B Y  4BPXA                291    0.175  37.291  -0.714   0.879   0.010   0.111
B G  4BPXA                292    0.082   6.414   0.298   0.858   0.002   0.139
E P  4BPXA                293    0.365  51.864   0.481   0.858   0.002   0.139
E W  4BPXA                294    0.347  83.574   0.578   0.923   0.002   0.076
B L  4BPXA                295    0.035   6.390  -0.421   0.923   0.002   0.076
B E  4BPXA                296    0.121  21.191  -0.296   0.923   0.002   0.076
B W  4BPXA                297    0.234  56.301   0.262   0.923   0.002   0.076
E E  4BPXA                298    0.274  47.903   0.284   0.923   0.002   0.076
B I  4BPXA                299    0.050   9.195   0.448   0.923   0.002   0.076
B M  4BPXA                300    0.085  16.988   0.224   0.938   0.007   0.055
B L  4BPXA                301    0.108  19.811   0.193   0.938   0.007   0.055
B Q  4BPXA                302    0.248  44.257  -0.011   0.938   0.007   0.055
B Y  4BPXA                303    0.217  46.287  -0.303   0.802   0.014   0.185
B C  4BPXA                304    0.102  14.293  -0.588   0.522   0.016   0.462
B F  4BPXA                305    0.175  35.042   0.294   0.181   0.016   0.803
B P  4BPXA                306    0.207  29.430  -0.397   0.053   0.043   0.903
E R  4BPXA                307    0.353  80.814   0.055   0.113   0.087   0.800
B L  4BPXA                308    0.081  14.794   0.041   0.056   0.142   0.802
E D  4BPXA                309    0.304  43.835   0.011   0.113   0.087   0.800
E I  4BPXA                310    0.259  47.933   0.128   0.307   0.165   0.527
E N  4BPXA                311    0.502  73.551   1.093   0.321   0.252   0.427
B V  4BPXA                312    0.043   6.624   0.040   0.428   0.171   0.402
B S  4BPXA                313    0.248  29.112   0.013   0.278   0.093   0.628
E K  4BPXA                314    0.559 114.883   0.673   0.354   0.048   0.598
E G  4BPXA                315    0.584  45.937  -0.718   0.268   0.043   0.689
B I  4BPXA                316    0.209  38.702   0.332   0.268   0.043   0.689
E N  4BPXA                317    0.387  56.657  -0.417   0.278   0.093   0.628
B H  4BPXA                318    0.234  42.583   0.664   0.321   0.252   0.427
B L  4BPXA                319    0.069  12.689  -0.516   0.352   0.332   0.316
B L  4BPXA                320    0.072  13.256   0.237   0.352   0.332   0.316
E K  4BPXA                321    0.355  73.024  -0.026   0.231   0.330   0.439
B S  4BPXA                322    0.110  12.857  -0.468   0.052   0.084   0.864
B P  4BPXA                323    0.190  26.961  -1.306   0.018   0.047   0.935
B F  4BPXA                324    0.277  55.594  -2.094   0.019   0.141   0.840
B S  4BPXA                325    0.123  14.404  -2.009   0.021   0.279   0.699
B V  4BPXA                326    0.104  16.000  -1.557   0.022   0.359   0.619
B H  4BPXA                327    0.196  35.689  -0.116   0.019   0.141   0.840
E P  4BPXA                328    0.386  54.830  -1.095   0.018   0.047   0.935
E K  4BPXA                329    0.497 102.295  -1.155   0.018   0.047   0.935
B T  4BPXA                330    0.155  21.554  -1.165   0.004   0.085   0.910
B G  4BPXA                331    0.108   8.476  -1.993   0.005   0.336   0.660
B R  4BPXA                332    0.222  50.746   0.218   0.003   0.718   0.279
B I  4BPXA                333    0.062  11.451  -0.022   0.001   0.900   0.099
B S  4BPXA                334    0.046   5.415  -0.382   0.001   0.959   0.040
B V  4BPXA                335    0.099  15.216   0.783   0.001   0.900   0.099
E P  4BPXA                336    0.279  39.590  -0.068   0.002   0.816   0.182
B I  4BPXA                337    0.065  11.951   0.702   0.004   0.420   0.576
E D  4BPXA                338    0.415  59.729   0.900   0.004   0.138   0.858
B L  4BPXA                339    0.156  28.637  -0.522   0.018   0.019   0.964
E Q  4BPXA                340    0.751 134.075   0.104   0.058   0.017   0.925
E K  4BPXA                341    0.558 114.698   1.466   0.115   0.016   0.868
B V  4BPXA                342    0.094  14.417  -0.231   0.115   0.016   0.868
E D  4BPXA                343    0.638  91.921  -0.418   0.115   0.016   0.868
E Q  4BPXA                344    0.640 114.233   1.129   0.058   0.017   0.925
B F  4BPXA                345    0.068  13.547   0.269   0.018   0.019   0.964
E D  4BPXA                346    0.357  51.458   0.587   0.005   0.015   0.979
B P  4BPXA                347    0.152  21.555  -0.856   0.018   0.019   0.964
E F  4BPXA                348    0.374  75.022  -0.687   0.018   0.047   0.935
E T  4BPXA                349    0.520  72.166  -0.147   0.053   0.043   0.903
B V  4BPXA                350    0.195  30.002   0.301   0.113   0.043   0.844
B P  4BPXA                351    0.127  17.979   0.343   0.257   0.016   0.727
B T  4BPXA                352    0.233  32.262   1.073   0.257   0.016   0.727
B I  4BPXA                353    0.053   9.861   0.964   0.858   0.002   0.139
E S  4BPXA                354    0.489  57.311   1.379   0.923   0.002   0.076
E F  4BPXA                355    0.411  82.387   1.591   0.970   0.001   0.030
B I  4BPXA                356    0.031   5.772   0.005   0.970   0.001   0.030
B C  4BPXA                357    0.174  24.416   0.495   0.970   0.001   0.030
E R  4BPXA                358    0.575 131.606   1.321   0.923   0.002   0.076
E E  4BPXA                359    0.261  45.562   1.007   0.923   0.002   0.076
B L  4BPXA                360    0.047   8.587   0.725   0.923   0.002   0.076
E D  4BPXA                361    0.469  67.626   0.191   0.858   0.002   0.139
E A  4BPXA                362    0.529  58.318   0.554   0.858   0.002   0.139
B I  4BPXA                363    0.161  29.841  -0.090   0.802   0.014   0.185
E S  4BPXA                364    0.300  35.137  -0.081   0.717   0.014   0.269
E T  4BPXA                365    0.504  69.905  -0.095   0.802   0.014   0.185
E N  4BPXA                366    0.541  79.188   0.027   0.802   0.014   0.185
E E  4BPXA                367    0.369  64.552  -0.300   0.802   0.014   0.185
E E  4BPXA                368    0.446  78.004  -0.424   0.717   0.014   0.269
E E  4BPXA                369    0.605 105.676  -0.724   0.717   0.014   0.269
E K  4BPXA                370    0.559 115.048  -0.085   0.622   0.015   0.363
E E  4BPXA                371    0.545  95.142  -0.528   0.522   0.016   0.462
E E  4BPXA                372    0.431  75.348  -1.032   0.430   0.016   0.555
E N  4BPXA                373    0.477  69.847  -1.353   0.354   0.048   0.598
E E  4BPXA                374    0.390  68.185  -1.345   0.354   0.048   0.598
E A  4BPXA                375    0.504  55.530  -1.500   0.354   0.048   0.598
E E  4BPXA                376    0.491  85.760  -0.424   0.354   0.048   0.598
E S  4BPXA                377    0.497  58.284  -1.204   0.455   0.046   0.498
E D  4BPXA                378    0.527  75.869  -0.295   0.455   0.046   0.498
E V  4BPXA                379    0.403  61.941  -1.246   0.430   0.016   0.555
E K  4BPXA                380    0.563 115.809  -0.593   0.430   0.016   0.555
E H  4BPXA                381    0.470  85.493  -0.070   0.455   0.046   0.498
E R  4BPXA                382    0.411  94.165  -0.057   0.354   0.048   0.598
B T  4BPXA                383    0.210  29.085  -1.440   0.354   0.048   0.598
E R  4BPXA                384    0.501 114.615   0.366   0.354   0.048   0.598
E D  4BPXA                385    0.589  84.904   0.126   0.278   0.093   0.628
B Y  4BPXA                386    0.214  45.817   0.878   0.278   0.093   0.628
E K  4BPXA                387    0.492 101.143  -0.356   0.278   0.093   0.628
E K  4BPXA                388    0.410  84.337   0.191   0.278   0.093   0.628
E T  4BPXA                389    0.423  58.642  -1.141   0.354   0.048   0.598
E S  4BPXA                390    0.302  35.441  -0.957   0.430   0.016   0.555
B L  4BPXA                391    0.125  22.814   0.147   0.600   0.003   0.397
E A  4BPXA                392    0.355  39.099   0.291   0.600   0.003   0.397
E P  4BPXA                393    0.479  67.942   0.429   0.782   0.003   0.216
B Y  4BPXA                394    0.071  15.258  -1.226   0.923   0.002   0.076
B V  4BPXA                395    0.070  10.713   0.352   0.975   0.003   0.022
E K  4BPXA                396    0.472  97.070   1.430   0.970   0.001   0.030
B V  4BPXA                397    0.146  22.425   1.100   0.970   0.001   0.030
B F  4BPXA                398    0.023   4.576   1.075   0.988   0.000   0.012
E E  4BPXA                399    0.327  57.127   1.059   0.988   0.000   0.012
E H  4BPXA                400    0.529  96.225   1.828   0.988   0.000   0.012
B F  4BPXA                401    0.090  18.063   0.942   0.970   0.001   0.030
B L  4BPXA                402    0.033   6.042   0.292   0.970   0.001   0.030
E E  4BPXA                403    0.566  98.845   1.929   0.970   0.001   0.030
E N  4BPXA                404    0.455  66.685   1.406   0.923   0.002   0.076
B L  4BPXA                405    0.074  13.586   0.883   0.923   0.002   0.076
E D  4BPXA                406    0.289  41.674   0.671   0.923   0.002   0.076
E K  4BPXA                407    0.617 126.896   1.476   0.923   0.002   0.076
E S  4BPXA                408    0.466  54.568   0.018   0.923   0.002   0.076
E R  4BPXA                409    0.319  73.051   0.097   0.858   0.002   0.139
E K  4BPXA                410    0.415  85.448   0.258   0.923   0.002   0.076
E G  4BPXA                411    0.581  45.748   0.543   0.923   0.002   0.076
E E  4BPXA                412    0.423  73.898   0.344   0.923   0.002   0.076
B L  4BPXA                413    0.220  40.319   0.638   0.970   0.001   0.030
E L  4BPXA                414    0.369  67.472   0.942   0.970   0.001   0.030
E K  4BPXA                415    0.541 111.222   1.020   0.923   0.002   0.076
E K  4BPXA                416    0.457  94.025   0.726   0.923   0.002   0.076
E S  4BPXA                417    0.476  55.752   0.314   0.858   0.002   0.139
E D  4BPXA                418    0.608  87.570   0.125   0.694   0.003   0.303
E L  4BPXA                419    0.483  88.401  -0.151   0.600   0.003   0.397
E Q  4BPXA                420    0.638 113.911   0.211   0.600   0.003   0.397
E K  4BPXA                421    0.533 109.618   0.347   0.321   0.003   0.675
E D  4BPXA                422    0.698 100.539  -0.288   0.181   0.016   0.803
E F  4BPXA                423    0.542 108.799  -1.258   0.003   0.003   0.994

Explain the output.  Go back.
```

---
